# Improving hand hygiene compliance leads to improved health outcome: an analysis

**DOI:** 10.1186/cc14162

**Published:** 2015-03-16

**Authors:** V Rao, A Datta, A Kar

**Affiliations:** 1 Medica Superspecialty Hospital, Kolkata, India

## Introduction

Hand hygiene is the single most effective but least practiced action in breaking the chain of transmission of microbes. Studies have shown a correlation between the compliance of hand hygiene and its impact on the health outcome.

## Methods

A quasi-experimental study was done in three level III ICUs of a tertiary care hospital in Kolkata (January to April 2014). Data were collected on existing hand hygiene compliance rate, ventilator-associated pneumonia (VAP) rate, catheter-related bloodstream infection (CRBSI) rate, catheter-related urinary tract infection (CAUTI) rate, standardized mortality ratio (SMR) and average ICU length of stay in the abovementioned units. Root cause analysis was done and interventions were developed to improve hand hygiene compliance and was implemented (July to October 2014). Comparison was done between preintervention and postintervention periods.

## Results

In the preintervention period (January to April 2014) the hand hygiene compliance among the caregivers was found to be 40%, VAP rate (8.77), CRBSI rate (3.42), CAUTI rate (5.27), SMR (1.14) and average ICU length of stay was 6 days ± 5.85 SD (median 4.5). Interventions were developed and implemented as follows: education and awareness - road shows; positive reinforcement; secret watch nurse; e-ICU - electronic surveillance; ring the bell once every hour - baseline hand hygiene; visual reminders; availability of alcohol-based hand rub, soap and water and sinks; random hand swabs; and compliance audits. In the postintervention period (July to October 2014) data showed a significant improvement in the hand hygiene compliance (75%). Further analysis showed an association with decrease in the incidence of VAP rate (4.71), CAUTI rate (3.51), CRBSI rate (2.65), SMR (1.05) and average ICU LOS 5.05 days ± 4.03 SD (median 4).

## Conclusion

Improved hand hygiene compliance can be attributed to decreased incidence of VAP, CRBSI, CAUTI, SMR and average ICU LOS. This does definitely impact the overall clinical outcome. However, continued surveillance of hand hygiene compliance and regular audits is of utmost importance to make the change sustainable.

